# Heterogeneity in cyber loss severity and its impact on cyber risk measurement

**DOI:** 10.1057/s41283-022-00095-w

**Published:** 2022-06-06

**Authors:** Martin Eling, Kwangmin Jung

**Affiliations:** 1 Institute of Insurance Economics, University of St. Gallen, Girtannerstrasse 6, 9010 St. Gallen, Switzerland; 2 Department of Industrial and Management Engineering, POSTECH (Pohang University of Science and Technology), Cheongam-ro 77, Nam-gu, Pohang, Gyeongbuk 37673 South Korea

**Keywords:** Operational risk, Cyber risk, Financial services industry, Tweedie model, C13, G32

## Abstract

We use the world’s largest publicly available dataset of operational risk to model cyber losses and show that the Tweedie model best fits the cyber loss severity in the financial industry. Three key determinants of loss severity are firm size, contagion risk and legal liability. We also measure the size of risk based on the estimation results and show a large degree of heterogeneity across financial firms. The results are particularly relevant with respect to the recent discussion on simplifying operational risk capital requirements and reiterate the importance of considering individual firm characteristics when modelling operational losses.

## Introduction

Operational risk is one of the key risks faced by the financial industry. The operational risk landscape has changed significantly over the last few years due to digital transformation and interconnected network systems, and such development has further intensified during the COVID-19 pandemic. These changes have led to cyber risk becoming one of the most threatening operational risks and a particularly challenging topic because of the lack of related data and potential contagion risk (Caporale et al. 2020; Uddin et al. 2020).^1^ Several studies have modelled the cyber loss process (see, e.g. Edwards et al. 2016; Eling and Jung 2018; Eling and Wirfs 2019; Wheatley et al. 2021), but none have focussed on the financial industry. This is problematic because research shows a large heterogeneity in cyber risk between financial and nonfinancial industries (Eling and Wirfs 2019); it also highlights that the financial industry is prone to large attacks given their distinct features, such as large lawsuits and potential systemic risk (Uddin et al. 2020; Aldasoro et al. 2022).

To fill this gap, we model cyber loss severity in the financial services industry considering the world’s largest publicly available dataset of operational risk (the SAS OpRisk database). We use a two-step approach with base model selection and regression analysis through generalized linear models (GLM). The Tweedie model best describes cyber losses; this model has been widely used in the actuarial field (see, e.g. Jørgensen and de Souza 1994; Quijano Xacur and Garrido 2015; Shi 2016; Peña-Sanchez 2019; Delong et al. 2021) but has not yet been considered in the context of operational risk and cyber risk. We develop three financial industry-specific factors and empirically document that firm size, contagion risk and legal liability are the key drivers of cyber loss severity in the financial industry.

Our results have important implications not only for cyber risk management but also for the regulation of operational risk in the financial industry in general. Until the most recent reform of operational risk measurement by the Bank for International Settlements (BIS) in 2016, the loss distribution approach (LDA), as part of the advanced measurement approaches (AMA), was a popular internal risk model. The LDA characterizes firms’ individual risks by loss frequency and severity (Jin et al. 2020; Zhu et al. 2021). The new reform requiring all banks to use the standardized measurement approach (SMA) simplifies the quantification method and is a model-free approach based on accounting information that mainly focuses on company size (BIS 2016). There is a debate about whether the SMA can accurately measure the required capital, and many academics are concerned that the SMA does not correctly reflect the heterogeneity of individual operational risks (see, e.g. Peters et al. 2016; Dionne 2019, p. 378).^2^ This study supports this point by claiming that operational risks in the financial industry should reflect the statistical features of firms’ individual risks.

Our study makes a threefold contribution to the literature. It is the first work to focus on cyber losses in the financial industry; second, it introduces the use of the Tweedie model for the calculation of cyber risk; and third, it makes an argument about a potential drawback of the Basel III reform. The paper is structured as follows. “Literature review and discussion of key factors” section reviews the relevant literature, followed by the methodology, which is provided in “Methodological background and model setup” section. “Data” section illustrates the data. The results and applications follow in “Results” section, and we conclude the paper in “Conclusion” section.

## Literature review and discussion of key factors

### Literature review

Operational risk management has been discussed extensively in the financial industry, especially how to accurately model this type of risk that is known for its heterogeneous features compared to other types of risks (such as market or credit risks). A financial institution could incur operational losses for various known and unknown reasons; a cyber breach could be one of these reasons. There is no established method for estimating operational risk exposure. Some researchers use risk events and loss data and analyse them using the value at risk (VaR) framework. However, challenges and pitfalls exist in measuring operational risk from loss data (Cope et al. 2009) because there is no standardized accounting approach or established record-keeping system to uniformly track losses from operational breakdowns across financial institutions globally.

In addition, financial institutions typically do not report operational loss data as a separate item in their audited financial statement or other disclosures. This means that financial institutions choose their own method for operational loss estimation. Hence, from an accounting perspective, loss data cannot be used as a reasonable proxy for operational risk. In regard to cyber risk, in some cases, loss information is complete (because of mandatory reporting requirements, for example, for data breaches in the U.S. and the European Union), whereas other types of risks are prone to the above reporting biases. For this reason, we do not claim that our estimation approach provides solutions to operational risk management in general. Our focus is rather narrow in the sense that we analyse drivers of cyber loss severity and predict the potential loss size based on them.

We review the distinct features of cyber risk events that the financial services sector may need to be aware of and how cyber operational losses have been modelled. Uddin et al. (2020) contribute to the discussion by analysing cybersecurity hazards and financial system vulnerability. They systematically review the literature to assess cybersecurity effects on operational costs of banks, their performance, operational risk-taking and cyber risk governance. Cyber risk significantly affects the costs of operational risk management for banks driven by the widespread digitalisation of banking operations. The authors identify that although financial regulators have suggested some nontechnical and technical guidelines for cyber risk governance, the underlying drivers of cyber risk events are not yet well understood. Our study contributes to filling this gap by proposing a two-step approach to address the statistical features and drivers of cyber loss severity.

Eling and Wirfs (2019) examine what drives the frequency and severity of cyber risk events. They use the same database of operational risk as that used in this study (SAS OpRisk) and analyse the data using the peaks-over-threshold model from extreme value theory. Economic losses are driven by the type of event and firm specifics such as country, industry or size. Heterogeneity among financial and nonfinancial firms is documented by including a dummy variable but is not further studied. The authors’ contribution is thus in line with that of the current study; however, we focus on the financial industry and suggest a new modelling approach (i.e. the Tweedie model).

Aldasoro et al. (2022) also investigate what factors drive cyber risk events using another large cyber risk-specific dataset from Advisen. They find that larger firms are more likely to have higher cyber costs than smaller firms, and events affecting multiple firms can lead to higher costs. Palsson et al. (2020) also study the features of cyber incidents using the Advisen dataset and determine that the financial sector is more exposed to cyber incidents and malicious risks can dominate overall cyber threats in most industry sectors.

Another set of studies examines how data breach losses can be modelled using the freely available Privacy Rights Clearinghouse (PRC) dataset. For example, Eling and Jung (2018) model the dependence structure of data breach losses by risk types and industries and provide potential risk measures at the aggregate level. Farkas et al. (2021) use a generalized Pareto approach to model extreme data breach claims, where loss frequency and severity are considered via regression trees. Our study can be differentiated from the above-mentioned literature as it proposes a two-step process to model cyber losses in which a factor analysis with an optimal distribution for the target variable is considered.

### Key factors affecting cyber loss severity

Based on their extensive review, Uddin et al. (2020) identify that the widespread application of cyber technology is a paradox because operational benefits come with the inherent risk of a cyber breach, and the effect is often unpredictable. Hence, it is essential to identify the reasons why an institution is more or less susceptible to cyber threats and which factors drive cyber loss severity to predict the potential amount of cyber losses. We focus on three factors that have been discussed in the literature as being critical and influential in explaining cyber risk events and loss amounts, namely, size, contagion risk and legal liabilities. The importance of these three factors may hold not only for the financial industry but also for other industries that are exposed to cyber risks. However, our focus is on the financial industry to discuss how the banking regulatory framework for operational risk can be further improved by taking into account the features of cyber operational risks.

The financial industry is known for being relatively more exposed to cyber risks (Uddin et al. 2020) and has the second largest average costs per breach event (after health care; see Ponemon Institute 2021). In this context, both industry studies and academic literature have documented that *firm size* is associated with the size of losses by cyber risk events (see, e.g. Ponemon Institute 2021; Aldasoro et al. 2022; Eling et al. 2022). While most results show a positive relationship, the elasticity of the firm size effect is shown to be rather low, which implies that large firms may not always be expected to face extremely large costs (Aldasoro et al. 2022). Eling et al. (2022) argue that this effect is due to economies of scale, regulatory compliance and the “wait-and-see” strategy of small firms potentially worsening network weaknesses.^3^ We thus consider firm size as one potential driver of loss severity.

The financial industry is also known for high *interconnectivity* with similar business models and similar IT systems between players; a security weakness in one party may cause a systemic impact on other parties. Relatedly, Eling et al. (2022) determine that a relatively weak cybersecurity system may lead to high total costs. This potential systemic impact of cyber risk events on the financial industry has also been discussed by Eisenbach et al. (2021). The complexity and high interconnectivity of the U.S. financial system may cause vulnerability in regard to cyberattacks. A systemic cyberattack could diminish a bank’s ability to serve its creditors. The authors empirically determine that the impairment of the five most active U.S. banks (i.e. the most interconnected parties) might result in spillover effects that impact an average of nearly 38% of their industry network. This finding reveals the concern that a systemic collapse caused by a catastrophic cyber event might be realized in the financial industry. Aldasoro et al. (2020) also show that the financial industry can have a large number of cyberattacks and thus be exposed to major threats to financial stability. Systemic risk is driven by large firms and particular business models that are more interconnected within the industry (Uddin et al. 2020). Based on the extensive literature, we analyse the level of contagion of cyber risk events and its impact on the loss amount.

In addition to size and contagion risk, *third-party liability* might significantly affect the total loss amount of cyber events. This is because cyber events not only cause direct losses but also spill over to clients, who then sue the attacked company. Such third-party liabilities might take up a significant amount of costs (Romanosky et al. 2019) and are covered by cyber-liability insurance, including legal liability, vicarious liability and network security liability (Biener et al. 2015). Legal liabilities represent defence and claims expenses and regulatory fines, whereas vicarious liabilities are caused by events occurring from outsourcing parties; network security liabilities primarily include costs from reinstatement and legal proceedings (Biener et al. 2015). Until recently, the majority of cyber claims resulted from general liability policies; however, claims tend to be rising for network security and privacy liability policies as well (McLeod 2015). Considering the potential significant impact of such liabilities on the total cyber costs, we disentangle a variable that addresses whether an event contains liability.

## Methodological background and model setup

### Tweedie process

To make the paper self-contained, we describe the key methodological steps of the suggested two-step estimation process. We first introduce the Tweedie process to be fitted to the empirical data in the first step. The Tweedie model is an efficient tool that can replace two-part model splitting loss frequency and severity by providing statistical features of two components in a single model (Tweedie 1984; Jørgensen and de Souza 1994). The probability density function of a Tweedie process shows the majority of its mass at zero and the rest highly skewed to the right; thus, it applies to datasets in which a large number of zeros and highly skewed non-zeros are mixed. As illustrated in Table 1, well-known discrete and continuous distributions used in a loss model can be represented by the Tweedie model as per its power parameter, $p\in \mathrm{R}$ (Ohlsson and Johansson 2010, Chap. 2).^4^

**Table 1:** Parameter and distribution types of the Tweedie process

Power parameter	Distribution type	Specific family
$p<0$	Continuous	–
$p=0$	Continuous	Gaussian
$0<p<1$	Nonexisting	–
$p=1$	Discrete	Poisson
$1<p<2$	Mixed, nonnegative	Poisson-Gamma (Compound)
$p=2$	Continuous	Gamma
$2<p<3$	Continuous	–
$p=3$	Continuous	Inverse Gaussian
$p>3$	Continuous	–

To describe the Tweedie distribution with its parameters, let us define ${y}_{j}$ by an i.i.d. response variable, which is independent of *N* following a Poisson distribution with mean $\lambda.$ Each ${y}_{j}$ follows a gamma distribution with a shape parameter of $\alpha$ and rate parameter of $\beta$; then, the summation of ${y}_{j},$ where $j=1,\ldots,N,$ is a Poisson sum of gammas (i.e. $S={\sum }_{j=1}^{N}{y}_{i}$). The Tweedie distribution facilitates mixing a peak point at $S=0$ and the other part $S>0.$ The peak of the distribution at $S=0$ represents the probability of zero loss; thus, $P\left(S=0\right)=P\left(N=0\right)={e}^{-\lambda }.$ Following Frees et al. (2016), we describe the other part where $S>0$ as follows:

1 $$P\left(S\le y\right)=\sum_{n=1}^{\infty }P\left(N=n\right)P\left({S}_{n}\le y\right), y\ge 0.$$

Thus, as Frees et al. (2016) define, the probability function of the Tweedie distribution is as follows:

2 $${f}_{S}\left(y\right)=\sum_{n=1}^{\infty }{e}^{-\lambda }\frac{{\lambda }^{n}}{n!}\frac{{\beta }^{n\alpha }}{\Gamma \left(n\alpha \right)}{y}^{n\alpha -1}{e}^{-y\beta },$$

where $\Gamma \left(\bullet \right)$ is the gamma function.

The parameters of the Poisson and gamma distributions incorporated in the Tweedie distribution can be addressed by the expectation of the variable *S* (i.e. $E\left(S\right)=\mu$), the scale parameter $\phi$ and the power parameter, *p*, as follows:

$$\lambda=\frac{{\mu }^{2-p}}{\phi \left(2-p\right)},$$

$$\alpha =\frac{2-p}{p-1},$$

$$\frac{1}{\beta }=\phi \left(p-1\right){\mu }^{p-1}.$$

A Tweedie process with a power parameter between 1 and 2 ($p\in \left({1,2}\right)$) is generally defined as a Poisson sum of a gamma distribution, which represents a compound loss process. For more detail on the specification of the Tweedie distribution, we refer to Jørgensen and de Souza (1994) and Ohlsson and Johansson (2006), where the mean and variance parameters and their relations with the power parameter are elaborated. By comparing the Tweedie model and other competitors, we determine the optimal model by the minimum Akaike information criterion (AIC) (Akaike 1973) and the acceptance of the null hypothesis of the Kolmogorov–Smirnov (K–S) goodness-of-fit test (Smirnov 1939).

### Variable description and the model setup

The second step of the process is to implement a regression-based model to investigate key drivers of cyber loss severity based on the fitted distribution from the first step. We first describe the variables to be used in the model. The target variable is the total loss (in million U.S. dollars) caused by a cyber risk event. The losses provided by the SAS database incorporate direct and indirect costs and consist of legal liability, regulatory action, asset damages, restitution, loss of recourse and write-down losses (Eling and Wirfs 2019).

As explanatory variables, we consider the three above-mentioned key variables as potentially affecting the amount of cyber losses. The first variable is firm size, which is represented by the annual revenue or the number of employees of victim organizations. The literature in the cyber risk context that uses different loss data from that used in our study has documented that the larger an institution exposed to cyber risk is, the larger the losses they likely to occur are (Romanosky 2016; Aldasoro et al. 2022; Jung 2021). This is, for example, because a larger organization tends to have a larger exposure (e.g. the number of computers counted for the number of employees and computerized assets) of vulnerability to cyberattacks.

We also take two variables into account for potential contagion effects, namely, events that either affect multiple firms or lead to multiple losses. Accounting for such effects with these variables is material to investigating the potential cyber systemic risk to which the financial industry can be significantly vulnerable. The third key variable included in the model is a variable that represents a firm exposed to liability risk. Identifying the impact of liability risk is significant because such risk can raise the total cost for victim organisations.

As control variables, we consider different risk types as binary variables, i.e. acts of people, system failures and failures in business processes. These risk types are based on the Basel classification of operational risks, where the external event risk is used as the base group in the model. Geographical location might also explain how severe cyber losses are. Developed countries with a high GDP, high levels of internet penetration and extensive internet use tend to have more potential victims of cyberattacks compared to their counterparts (Namestnikov 2012). To address potential heterogeneity in cyber exposure in the geographical aspect, we consider three geographical variables, namely, Asia, Europe and North America. Observations in other areas are categorized as “others” and used as the base group for this type of information in the model. Table 2 shows the target variable (total loss) and explanatory variables used in the second step of the estimation process.

**Table 2:** Variable description

Variable	Description	Type
Total loss	The size of total loss in million dollar by a cyber risk event	Numeric
Firm size	The annual revenue or the number of employees	Numeric
Multiple firms affected	Risk events affecting multiple firms	Binary
Multiple losses	Risk events generating multiple losses	Binary
Liability	Cases where breached entities paid legal liabilities	Binary
People	Risk events caused by acts of people, consisting of external and internal fraud	Binary
System	Risk events caused by business disruption and system failures	Binary
Process	Risk events caused by failures in internal business processes, consisting of failures in clients, products and business practices, employment practices and workplace safety, execution, delivery and process management	Binary
Asia	Risk events occurring in Asia	Binary
Europe	Risk events occurring in Europe	Binary
North America	Risk events occurring in North America	Binary

For the firm size as a key variable in our model, in Table 5, we consider two proxy variables, namely, the amount of annual revenue and the number of employees, to see the impact of the firm size on the determination of cyber loss amount

Using all of these variables, we construct a regression-based model to investigate the impacts of key variables on the cyber losses amount. We apply the optimal distribution of cyber losses to model the response variable of the GLM in a matrix form for conciseness as follows:

3 $$g\left({\mu }_{i}\right)={\mathbf{\rm X}}_{i}^{^{\prime}}{\varvec{\beta}},$$

where $g\left(\bullet \right)$ is a known function called a link, ${\mu }_{i}$ is the mean of the response ${y}_{i},{\mathbf{X}}_{i}$ is a matrix expression of explanatory variables described in Table 2 and ${\varvec{\beta}}$ is a vector of coefficients. The underlying distribution of the response variable ${y}_{i}$ can be specified in the exponential family, where discrete (e.g. Poisson), continuous (e.g. normal, gamma) and mixture (Tweedie) distributions are included (Frees 2009, Chap. 13). In this study, we aim to model cyber loss severity in the continuous form, where the distribution of the response variable belongs to the exponential family. We use the fitted values of the model to simulate aggregate cyber losses and estimate risk measures.^5^

## Data

**Fig. 1:**
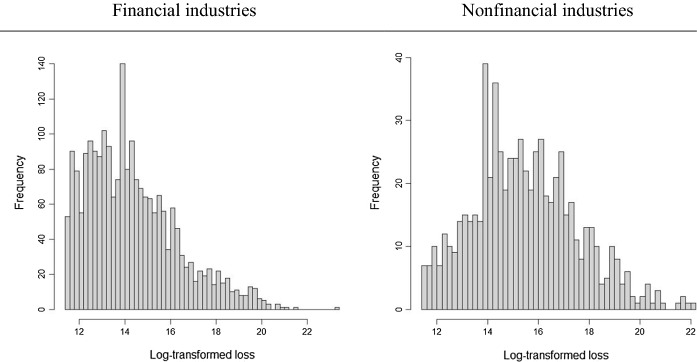
Histograms of log-transformed (natural logarithm) total cyber loss in financial (left) and nonfinancial (right) industries

We consider the SAS operational risk database,^6^ which has data collected from March 1984 to May 2021 and provides loss events above $100,000.^7^ The database includes 37,652 operational loss events worldwide, which makes it the world’s largest and most comprehensive repository of publicly available information on operational losses. We use 2,852 observations related to cyber risk, following the text mining presented in Eling and Wirfs (2019).^8^ We differentiate the financial services industry (e.g. banking, insurance, card issuers) from nonfinancial industries. This selection leads to 2186 (666) cyber loss events from the (non)financial industry.^9^ The data include information on risk types, dates of events, regions, firm-specific factors (e.g. revenue, assets, shareholder equity, the number of employees), loss size and contagion effects (e.g. whether multiple cases are affected or not). The target variable is longer-tailed for the financial industry than for nonfinancial industries (see Fig. 1).

Firm size is represented by either revenue or number of employees; to avoid multicollinearity between two firm size proxies, we test these variables in two different models. Descriptive statistics are shown in panels A and B of Table 3 for financial and nonfinancial industries, respectively.^10^ Firm size and average loss size are larger for nonfinancial firms; however, the variation and the maximum size of cyber losses are larger for financial firms. This might mean that although nonfinancial firms might experience larger cyber losses on average, extreme cases are more likely to occur in financial firms.

**Table 3:** Descriptive statistics of cyber risk data

	Mean	SD	Min	Median	Max
*Panel A: Financial industries (N* = *2,186)*					
Total loss	24.953m	280.712m	0.100m	1.100m	12,180.700m
Revenue	24,599.428m	35,827.167m	− 0.830m	5,955.000m	256,776.000m
Employees	61,327.647	87,270.541	0.000	20,952.000	496,698.000
Multi.Firm	0.193	0.395	0.000	0.000	1.000
Multi.Loss	0.116	0.321	0.000	0.000	1.000
Liability	0.086	0.280	0.000	0.000	1.000
People	0.686	0.464	0.000	1.000	1.000
System	0.040	0.197	0.000	0.000	1.000
Process	0.272	0.445	0.000	0.000	1.000
Asia	0.208	0.406	0.000	0.000	1.000
Europe	0.245	0.430	0.000	0.000	1.000
N. America	0.472	0.499	0.000	0.000	1.000
*Panel B: Nonfinancial industries (N* = *666)*					
Total loss	57.094m	275.849m	0.100m	4.240m	4,000.000m
Revenue	30,221.115m	67,206.679m	0.000m	6,783.850m	838,227.000m
Employees	68,793.249	147,117.996	1.000	19,809.000	2,100,000.000
Multi.Firm	0.117	0.322	0.000	0.000	1.000
Multi.Loss	0.177	0.382	0.000	0.000	1.000
Liability	0.317	0.466	0.000	0.000	1.000
People	0.536	0.499	0.000	1.000	1.000
System	0.078	0.268	0.000	0.000	1.000
Process	0.347	0.476	0.000	0.000	1.000
Asia	0.141	0.348	0.000	0.000	1.000
Europe	0.188	0.391	0.000	0.000	1.000
N. America	0.631	0.483	0.000	1.000	1.000

Employees: the number of employees. People: external and internal fraud. System: business disruption and system failures. Process: failures in business processes. Multi.Firm: events affecting multiple firms. Multi.Loss: events causing multiple losses. Africa: events occurring in Africa. Asia: events occurring in Asia. Europe: events occurring in Europe. N. America: events occurring in North America. Liability: whether a victim experienced litigation. The unit of variables, “Total loss” and “Revenue” is in millions of US-dollars ($), thus “m” stands for million

We implement a two-step approach to investigate the loss severity of financial and nonfinancial industries and to estimate potential loss size based on cyber operational risk. This two-step approach consists of finding an optimal distribution for univariate loss severity and then predicting the loss amount based on the generalized linear model with the optimal distribution identified in the first step. For the first step, we consider the Tweedie model and five other nonnegative distributions (i.e. lognormal, gamma, Weibull, Cauchy and lognormal-GPD of the peaks-over-threshold method) that have been widely used in operational loss and cyber risk modelling (see, e.g. Shevchenko 2010; Eling and Jung 2018; Eling and Wirfs 2019).

## Results

**Fig. 2:**
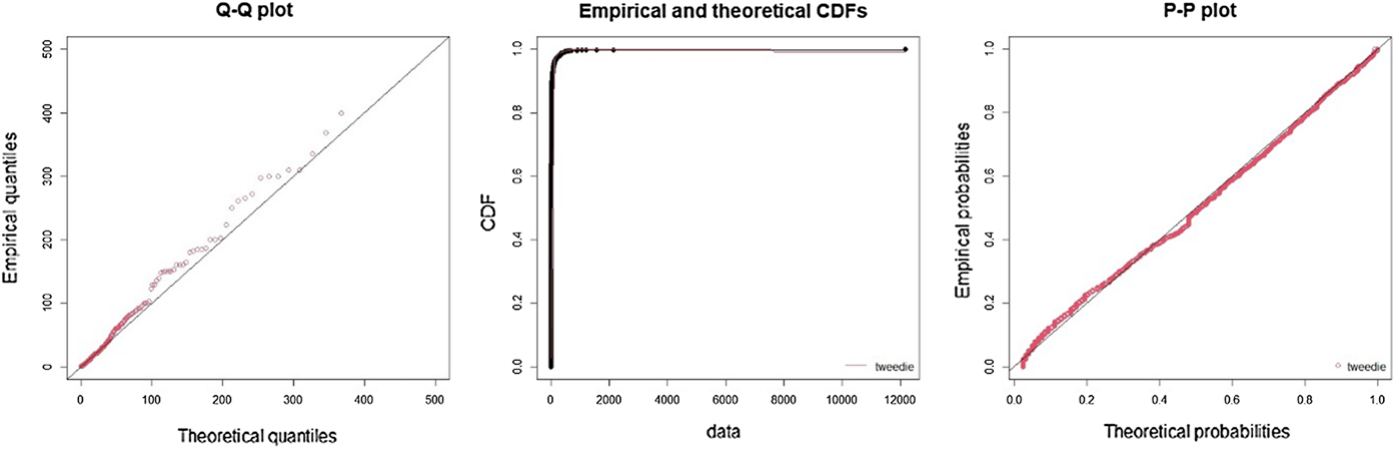
Graphical diagnosis of the Tweedie fit for the first step. The *Q*–*Q* plot, CDF and *P*–*P* plot are displayed in turn from the left

Table 4 shows the result of the model selection step for cyber loss severity by testing six competitive models with AIC values and K–S goodness-of-fit test statistics. We find that the Tweedie distribution best fits the cyber losses for both the financial and nonfinancial industries with the lowest AIC value and the acceptance of the null hypothesis of the K–S test. This finding is supported in Fig. 2, where good fits for the Tweedie are observed in the Q-Q, CDF and P-P plots. We then apply the Tweedie distribution to the response variable of the GLM approach in Table 5, where we examine key factors that drive the size of cyber losses.

**Table 4:** Optimal base model for cyber loss severity (step 1)

Distribution	Financial industry			Nonfinancial industry		
	Loglik	AIC	K–S test	Loglik	AIC	K–S test
Tweedie	**− 5,479.53**	**10,965.07**	**0.033**	**− 2,495.44**	**4,996.88**	**0.040**
Lognormal	**− **5,694.71	11,393.42	0.078	**− **2,506.99	5,017.97	0.050
Gamma	**− **6,714.51	13,433.01	0.242***	**− **2,735.24	5,474.48	0.203***
Weibull	**− **6,113.25	12,230.50	0.170***	**− **2,597.52	5,199.03	0.099
Cauchy	**− **6,913.47	13,830.94	0.305***	**− **2,928.50	5,861.01	0.306***
Lognorm-GPD	**− **5,628.97	11,265.95	0.062	**− **2,504.79	5,017.58	0.046

*,**,*** indicate that the p value is less than the significance levels 10%, 5% and 1%, respectively. We use the R package tweedie for the estimation. To avoid abnormal effects of extreme outliers, we truncate observations above the 99.5% quantile. Truncation to set an upper bound of an extreme operational loss data can be considered to deal with heavy-tail phenomenon and properly estimate the expected shortfall (Cirillo and Taleb 2016). We also check truncation at the 99% level, which does not change the optimal model selection. The bold indicates the best fit distribution based on AIC and goodness-of-fit test result

**Table 5:** GLM results using the optimal Tweedie base model (step 2)

	Dependent variable: total loss amount ($ million)							
	Financial services industries				Nonfinancial industries			
	Firm size = Revenue		Firm size = Num.Employees		Firm size = Revenue		Firm size = Num.Employees	
	Full model	Nested model	Full model	Nested model	Full model	Nested model	Full model	Nested model
Ln(Revenue)	0.0009 (0.0009)	0.0027*** (0.0009)			0.0033 (0.0021)	0.0032 (0.0021)		
Num.Employees			5.781e−08* (3.258e−08)	6.710e−08** (3.239e−08)			4.029e−09 (3.690e−08)	− 8.356e−10 (3.676e−08)
Multi.Firm	0.0304*** (0.0070)	0.0290*** (0.0070)	0.0296*** (0.0069)	0.0289*** (0.0071)	0.0399** (0.0167)	0.0365** (0.0164)	0.0438*** (0.0167)	0.0406** (0.0165)
Multi.Loss	0.0890*** (0.0089)	0.0984*** (0.0089)	0.0888*** (0.0088)	0.1005*** (0.0088)	0.0310** (0.0143)	0.0273* (0.0141)	0.0319** (0.0143)	0.0281** (0.0141)
Liability	0.0665*** (0.0104)	0.0678*** (1.829)	0.0676*** (0.0104)	0.0701*** (0.0102)	0.0416*** (0.0128)	0.0383*** (0.0118)	0.0418*** (0.0128)	0.0388*** (0.0118)
People	− 0.0748 (0.0724)		− 0.0761 (0.0723)		− 0.0347* (0.0291)		− 0.0379 (0.0292)	
System	− 0.0148 (0.0736)		− 0.0145 (0.0735)		0.0105 (0.0344)		0.0099 (0.0346)	
Process	− 0.0469 (0.0725)		− 0.0478 (0.0725)		− 0.0342 (0.0302)		− 0.0369 (0.0303)	
Asia	0.0056 (0.0119)		0.0046 (0.0118)		− 0.0496* (0.0296)		− 0.0454 (0.0295)	
Europe	0.0230** (0.0116)		0.0210* (0.0117)		− 0.0625** (0.0294)		− 0.0573* (0.0293)	
N. America	− 0.0196* (0.0109)		− 0.0220** (0.0110)		− 0.0530** (0.0278)		− 0.0476* (0.0277)	
Constant	2.6826*** (0.0754)	2.5754*** (0.0200)	2.7010*** (0.0730)	2.6290*** (0.0038)	2.7211*** (0.0566)	2.6442*** (0.0462)	2.7910*** (0.0351)	2.7140*** (0.0074)
Tweedie parameter	1.9	1.9	1.9	1.9	1.9	1.9	1.9	1.9

This table shows estimation results of the Tweedie-GLM for cyber loss events in financial services and nonfinancial industries. The estimations for both industries are presented using either firm’s annual revenue or the number of employees as a proxy for firm size. The optimal parameter of power for the Tweedie model (= *p*) falls into the range between 1 and 2, indicating a Poisson-distributed sum of gamma distribution as a compound process. The response variable is the log-transformed total loss amount caused by cyber risk events and the explanatory variables are the firm size, risk types, contagion risk, regions and whether legal liability was paid. For the Tweedie parameter, we test the nonnegative range (0 to 3) with 0.1 increments. The figures in the parentheses of estimate cells are standard errors and “e” stands for exponentiation with base ten. *,**,*** indicate that the *p* value is less than the significance levels 10%, 5% and 1%, respectively. We use the R package tweedie for this estimation

In Table 5, we present the Tweedie-GLM results in three categories: (1) the full model with the full set of variables vs. a nested model with our three key factors,^11^ (2) annual revenue vs. the number of employees as firm size proxy and (3) financial industry vs. nonfinancial industry. A compound process with the power parameter (*p*) between 1 and 2 turns out to be fitted best for our dataset in all models (1.9 in Table 5).

We find that firm size represented by either the annual revenue or the number of employees is mostly significant for the financial firms in explaining the size of total loss by cyber operational risk events. In particular, the statistical significance of the firm size variables is stronger for the nested models. The signs of the coefficients of firm size variables are positive, which implies that cyber costs increase with firm size. The results also confirm the literature claiming that the elasticity of the firm size effect can be low. Our result shows that, for example, under the nested model using annual revenue, a 1% increase in log-revenue (thus, a larger size of increase in revenue without log-transformation) may lead to only a 0.27% increase *on average* in the cyber cost (= exp(0.0027)), ceteris paribus. That is, the increase in cyber costs may not be too sensitive to firm size, which can result from economies of scale, regulatory compliance and the underinvestment of small firms in cybersecurity (Eling et al. 2022).

We also determine that the contagion effects that affect multiple firms and cause multiple losses through a single event are positive and significant for the financial industry in explaining the total costs. This result supports the documented findings of the literature that contagious cyber events can worsen the financial impacts on victim organizations. In particular, our finding is in line with Eling et al. (2022) that a weak cybersecurity potentially increases risk; contagion risk can have significant financial impacts on large-scale events.

As last key factor of cyber costs, financial firms also appear to have significant legal payments; hence, they tend to face higher legal liability costs. This evidence is in line with Bouveret (2018), who argues that the higher risks faced by the financial industry may result from a loss of trust and confidence due to the utilized business model that has capital management and monetary transactions at its core. This might be associated with legal actions by customers when cyber risks (such as data breaches) affect their financial accounts or systems.

For the other variables, we note that the European affiliation of financial firms may lead to an increase in cyber cost, whereas firms affiliated with North America exhibit lower costs. However, when considering the nonfinancial industry case as a benchmark, both coefficients of European and North American affiliation are negative and significant. Although we have no explicit evidence on this finding between European and North American firms, one possible explanation might be the regulation of cyber event disclosure. As Gordon et al. (2018) note, the 2011 U.S. Securities and Exchange Commission (SEC) disclosure guidance has pushed North American firms to report their cybersecurity risks and incidents, which may motivate them to enhance cybersecurity as a significant element of their internal controls. Last, the comparison with nonfinancial industries as a benchmark helps us understand that contagion and legal risks may still significantly affect other types of entities that are exposed to cyber risks, as we argue in “2.2” section. In Appendix 2, we further investigate how the impacts of firm-specific factors can be above certain thresholds for extreme loss events (i.e. 56%, 90% and 95%), where we observe mostly identical results to those found in Table 5.

We use the estimation results of Table 5 to predict how vulnerable firms are to potential cyber events and to illustrate the potential heterogeneity across firms. In Table 6, we generate the Tweedie distributions by calibrating Tweedie parameters from the fitted models for financial firms. Risk measures from another GLM-based model using gamma for a continuous response variable, those from three univariate models (lognormal, lognormal-GPD and empirical) and those for other operational risks (i.e. noncyber) from three univariate models are considered as benchmarks.^12^ We differentiate potential losses by considering whether contagion risk and liability payment are present.^13^ We simulate the distributions with 100,000 iterations^14^ and consider value-at-risk (VaR) and expected shortfall (ES) at the 99% quantile, which are both used in regulatory frameworks (e.g. Basel III and the Swiss Solvency Test).

**Table 6:** Loss estimates of cyber operational risk events in the financial industry

	With CR and LP		Without CR and LP	
	VaR 99%	ES 99%	VaR 99%	ES 99%
*Panel A: Optimal Tweedie model vs. gamma model as a benchmark under the two-step process*				
Fitted model with Tweedie	103.619	121.887	47.910	52.181
Gamma as a base (Benchmark I)	116.684	141.484	48.228	52.621

	Cyber risks		Other OpRisks (noncyber)	
	VaR 99%	ES 99%	VaR 99%	ES 99%
*Panel B: Benchmark II (univariate models and other OpRisks, both with CR and LP)*				
Lognormal	91.596	142.998	159.756	226.421
Lognormal-GPD	84.494	198.086	186.619	464.079
Empirical	114.320	137.393	165.000	190.066

We consider hypothetical European financial firms facing cyber operational risks. The loss estimates for value-at-risk (VaR) and expected shortfall (ES) at the 99% percentile are derived from cases that include contagion risk (CR) and liability payment (LP) as covariates or not. Three benchmark cases are considered; one is the loss estimates under the typical gamma-GLM approach in panel A, the second is the loss estimates for financial firms facing other operational risk under the fitted approach (Tweedie-GLM), and the last is the loss estimates with a typical LDA that has been widely used in operational risk modelling as part of the AMA and implicitly contains the impacts of CR and LP from the loss series. We do not consider a benchmark for the currently discussed SMA because it is a nonmodel-based method (BIS 2016). To avoid abnormal effects of extreme outliers in estimations in panel B, we again winsorize observations above the 99.5% quantile

The results in Table 6 demonstrate that contagion risk and legal liabilities can impose a heavy burden on victims. Financial firms with high levels of interconnectivity are more likely to be exposed to large loss amounts going beyond $100 m which is more than four times the average loss amount of $ 24.953 m documented in Table 3.^15^ Moreover, cyber risk events could generate huge liability costs for victims. We observe that the loss estimates of the Tweedie-GLM are slightly smaller than those of the gamma GLM. For univariate models that do not consider firm characteristics, our loss estimates are smaller for expected shortfall than those of lognormal and lognormal-GPD.

We also find that other operational losses are larger than those of cyber losses under the univariate models. This finding is in line with Eling and Wirfs (2019), who find that the distribution of noncyber operational losses is heavier-tailed than that of cyber losses. Overall, our application results indicate that neglecting heterogeneity from interconnectivity and legal liability might lead to firms being in trouble with regard to capital management, thereby increasing their likelihood of insolvency. Moreover, insurers that provide corporate solutions against cyber risks may face potential extreme claims of cyber risks if such heterogeneity in cyber risks is not fully addressed in their underwriting process.

Although we do not provide a direct comparison of our results with the SMA (Basel III), our findings provide directions for improving operational risk measurement, particularly with regard to cyber operational risks. Peters et al. (2016) outline the flaws of the SMA as follows: it introduces capital instability, reduces risk responsivity, induces risk-taking, fails to utilize the range of data sources, fails to provide risk management insight and can be a super-additive capital calculation. Based on their results, the authors argue that the SMA cannot be considered as an alternative to AMA models (and instead propose the standardization of AMA models). Our results illustrate many of these general points for the case of cyber risk and suggest that a standardized AMA model might better reflect the operational risk environment. The consideration of pure volume-based indicators from the annual report (as done in the SMA, mainly reflecting business size)^16^ does not well reflect the risk sensitivity and might create problematic incentives, at least in regard to cyber risk.

Specifically, the potential contagion risk that can be heterogeneous for banks due to their interconnectedness may not be fully addressed in the SMA formulation. It is difficult to identify what factors in the SMA can incorporate potential losses (or costs) from interconnectivity in the network environment. For example, IT-related administrative costs or outsourcing IT supplies are not included in the profit and loss items of the SMA formulation (BIS 2016, Annex 1). Our findings illustrate that banks with higher contagion risks and higher legal risks should have higher capital for cyber risks. Thus, considering the increasing impacts of cyber risks and the increasing dependency on digital transformation in the financial industry, regulators may need to take the impact of interconnectivity in the financial systems on operational losses into account.

## Conclusion

We propose a two-step approach to modelling large cyber losses with a systematic, statistical process. We use cyber risk-related loss data from the world’s largest publicly available database of operational risk to find an optimal base model for loss severity and the key determinants of such losses for the financial services industry. The Tweedie model best explains the statistical features of large cyber losses. The key drivers of determining cyber losses of financial firms are firm size, legal liability and contagion effects that cause multiple losses from a single event. Thus, contagion and legal risk are important features of cyber events that must be properly managed. Additionally, cyber risk management must be aligned with firm size. We also show the heterogeneity across firms by applying our model to estimate risk measures and find that contagion risk and liability payments can nearly double the loss estimates. This finding implies that a heterogeneous level of interconnectivity and potential liability should impact the size of the capital requirement if financial firms want to be able to fully cover potential extreme cyber losses. These variables also need to be reflected in insurance prices if a respective amount of coverage is to be offered.

The heterogeneity across firms sheds some critical light on the decision of financial regulators to simplify the operational risk capital requirement; it also shows the importance of considering firm characteristics and of taking a loss distribution-based approach to the modelling of operational risks by investigating cyber operational losses. For example, the potential contagion risk that is currently not considered in the reform of operational risk measurement may have heterogeneous impacts on firms’ cybersecurity capacity and interconnectivity, and this risk may cause considerable losses that heavily affect firms’ capital management. Although the LDA is no longer valid for operational risk measurement under the Basel framework, a potential way to improve operational risk measurement is to standardize a stepwise approach to consider such heterogenous features under the GLM-based analysis. Traditional actuarial rate-making may help to achieve this; however, from the financial regulatory perspective, one may need to consider the extent to which heterogeneous firm-specific factors affecting operational loss events can be allowed.

## Figures and Tables

**Fig. 3:**
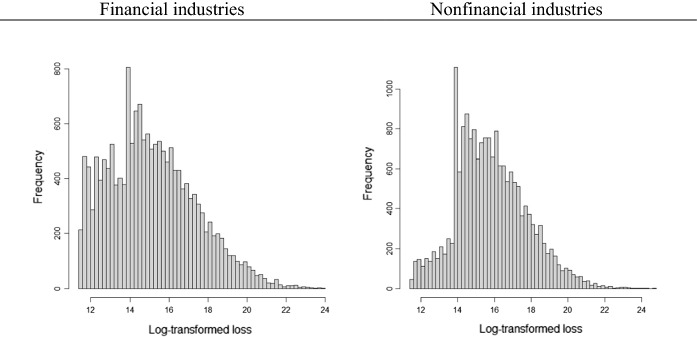
Histograms of log-transformed total operational loss in financial (left) and nonfinancial (right) industries

## Appendix 1: Summary statistics of operational risk data

See Table 7 and Fig. 3.

**Table 7:** Descriptive statistics of operational risk (noncyber) data

	Mean	SD	Min	Median	Max
*Panel A: financial industries (N = 17,504)*					
Total loss	72.668m	561.719m	0.100m	2.960m	23,240.000m
Revenue	25,346.254m	41,474.417m	− 28,575.300m	8,096.220m	2,655,002.800m
Employees	59,608.973	86,295.120	0.000	21,729.000	745,000.000
Multi.Firm	0.337	0.473	0.000	0.000	1.000
Multi.Loss	0.098	0.298	0.000	0.000	1.000
Liability	0.346	0.476	0.000	0.000	1.000
People	0.412	0.492	0.000	0.000	1.000
System	0.000	0.013	0.000	0.000	1.000
Process	0.579	0.494	0.000	1.000	1.000
Asia	0.145	0.352	0.000	0.000	1.000
Europe	0.226	0.418	0.000	0.000	1.000
N. America	0.578	0.494	0.000	1.000	1.000
*Panel B: nonfinancial industries (N = 18,152)*					
Total loss	80.346m	787.793m	0.100m	6.000m	58,000.000m
Revenue	41,649.617m	113,107.957m	− 1,415.500m	8,469.845m	2,655,002.800m
Employees	62,760.136	149,753.541	0.000	21,000.000	2,300,000.000
Multi.Firm	0.250	0.433	0.000	0.000	1.000
Multi.Loss	0.121	0.327	0.000	0.000	1.000
Liability	0.650	0.477	0.000	1.000	1.000
People	0.088	0.284	0.000	0.000	1.000
System	0.009	0.096	0.000	0.000	1.000
Process	0.660	0.474	0.000	1.000	1.000
Asia	0.122	0.328	0.000	0.000	1.000
Europe	0.200	0.400	0.000	0.000	1.000
N. America	0.642	0.479	0.000	1.000	1.000

Employees: the number of employees. People: external and internal fraud. System: business disruption and system failures. Process: failures in business processes. Multi.Firm: events affecting multiple firms. Multi.Loss: events causing multiple losses. Africa: events occurring in Africa. Asia: events occurring in Asia. Europe: events occurring in Europe. N. America: events occurring in North America. Liability: whether a victim experienced litigation. The unit of variables, “Total loss” and “Revenue” is in millions of US-dollars ($)

## Appendix 2: An analysis of large-scale cyber losses

Many operational (and cyber) risk studies focus on modelling large losses by taking a threshold-based approach that investigates observations above a certain threshold (see, e.g. Chavez-Demoulin et al. 2016; Eling and Wirfs 2019). This is because large losses are of primary interest for industries in terms of capital management; thus, understanding extreme loss events is expected to help firms improve their financial stability.

We examine the impact of firm-specific factors on large loss events by considering three thresholds. The quantile of 56% is used, as it is in Eling and Wirfs (2019), where this quantile value is found to be optimal for the extreme value model (i.e. peaks-over-threshold model) when using the same dataset used in this study. We also consider the 90% and 95% quantiles. One may consider more extreme quantiles (e.g. 99% or 99.5% in order to be aligned with financial regulatory frameworks, i.e. Basel III or Solvency II); however, in this case, the sample size is too small to obtain robust results. To align with the main approach in “Results” section, we again use the Tweedie-GLM approach for extreme cyber loss severity; here, we consider the annual revenue as a proxy for firm size.

Table 8 illustrates the results of both models with our full set of variables and nested models with our three key factors. We see that the key variables of financial firms for extreme events above the threshold of the 56% quantile have significant impacts on the total loss amount, particularly in the nested models. Specifically, the coefficients of variables for contagion risks (multi firms and multi losses) are positive and significant for the financial industry in explaining the loss amount. For more extreme quantiles (i.e. 90% and 95%), we observe signs of variables that are mostly identical to those found in Table 5 and those for the 56% quantile, but the impact is not significant. This outcome might be due to the relatively small sample size and high variation of the estimators.

**Table 8:** Results of analysing extreme cyber losses

	Dependent variable: Ln(Total loss amount) above each threshold											
	Financial services industries						Nonfinancial industries					
	Quantile 56%		Quantile 90%		Quantile 95%		Quantile 56%		Quantile 90%		Quantile 95%	
	Full model	Nested model	Full model	Nested model	Full model	Nested model	Full model	Nested model	Full model	Nested model	Full model	Nested model
Ln(Revenue)	0.0007 (0.0010)	0.0020** (0.0010)	0.0013 (0.0018)	0.0019 (0.0017)	0.0003 (0.0023)	0.0009 (0.0021)	0.0015 (0.0020)	0.0017 (0.0020)	0.0030 (0.0033)	0.0017 (0.0033)	0.0043 (0.0036)	0.0033 (0.0037)
Multi.Firm	0.0207*** (0.0069)	0.0195*** (0.0070)	0.0041 (0.0078)	0.0085 (0.0082)	− 0.0078 (0.0090)	− 0.0050 (0.0089)	0.0224 (0.0139)	0.0149 (0.0138)	− 0.0281 (0.0191)	− 0.0255 (0.0184)	− 0.0100 (0.0258)	− 0.0246 (0.0246)
Multi.Loss	0.0573*** (0.0078)	0.0661*** (0.0078)	0.0136* (0.0082)	0.0900 (0.0082)	0.0059 (0.0100)	0.0019 (0.0095)	0.0194* (0.0117)	0.0170 (0.0114)	− 0.0152 (0.0168)	− 0.0165 (0.0162)	− 0.0089 (0.0197)	− 0.0088 (0.0207)
Liability	0.0310*** (0.0090)	0.0352*** (0.0088)	0.0033 (0.0087)	0.0030 (0.0090)	− 0.0029 (0.0102)	− 0.0046 (0.0098)	− 0.0165 (0.0111)	− 0.0144 (0.0100)	− 0.0014 (0.0173)	0.0029 (0.0150)	− 0.0200 (0.0206)	0.0056 (0.0185)
People	− 0.2239** (0.0859)		− 0.0834 (0.0520)		− 0.0214 (0.0440)		0.0229 (0.0230)		− 0.0202 (0.0387)		0.0182 (0.0345)	
System	− 0.1712** (0.0867)		− 0.0746 (0.0529)		− 0.0325 (0.0449)		0.0498* (0.0275)		− 0.0391 (0.0429)		0.0064 (0.0472)	
Process	− 0.1925** (0.0860)		− 0.0467 (0.0520)		− 0.0054 (0.0435)		0.0377 (0.0238)		− 0.0066 (0.0400)		0.0604 (0.0374)	
Asia	0.0200 (0.0122)		0.0383* (0.0205)		0.0111 (0.0319)		0.0080 (0.0252)		0.0222 (0.0400)		0.0321 (0.0346)	
Europe	0.0184 (0.0119)		0.0167 (0.0198)		0.0053 (0.0315)		− 0.0287 (0.0251)		− 0.0233 (0.0416)		− 0.0194 (0.0404)	
N. America	− 0.0015 (0.0115)		− 0.0030 (0.0200)		− 0.0096 (0.0317)		− 0.0197 (0.0233)		− 0.0230 (0.0389)		0.0219 (0.0357)	
Constant	2.9393*** (0.0891)	2.7068*** (0.0220)	2.9279*** (0.0663)	2.8631*** (0.0378)	2.9621*** (0.0715)	2.9378*** (0.0458)	2.8047*** (0.0523)	2.8152*** (0.0458)	2.9365*** (0.1016)	2.9339*** (0.0740)	2.8666*** (0.1047)	2.9317*** (0.0825)

This table shows the estimation results of the Tweedie-GLM for extreme loss events in financial services and nonfinancial industries. The estimations for both industries are presented using firm’s annual revenue as a proxy for the firm size. The response variable is the log-transformed total loss amount caused by cyber risk events and the explanatory variables are the firm size, risk types, contagion risk, regions and whether a victim paid legal liability. The sample sizes for 56, 90 and 95% quantile models are 964, 219 and 110 for the financial industry, respectively, and 293, 67 and 34 for the nonfinancial industry, respectively. The figures in the parentheses of estimate cells are standard errors. *,**,*** indicate that the *p* value is less than the significance levels 10%, 5% and 1%, respectively

## Footnotes

1 The frequency and severity of cyber losses have increased over the last decade, with a structural break in loss severity present since 2014 (Jung, 2021).

2 Data from the European Banking Authority (EBA) show that the reform of operational risk measurement in Basel III will increase the required capitals of European banks for operational risk by 40.5% on average from the level at the end of 2020 (Migliorato 2021). This implies that the nonmodel based, homogeneous approach of the new reform may burden banks in regard to their capital management.

3 According to Eling et al. (2022), economies of scale may result from the underinvestment of small firms in cyber security systems due to their limited budgets and difficulties in acquiring skilled personnel. Additionally, regulatory compliance such as the Sarbanes–Oxley Act of 2002 and the 2011 U.S. Securities and Exchange Commission disclosure guidance (with requirements for firms to disclose cyber incidents) force firms to provide reliable financial reports with secure information systems. Finally, the anecdotal “wait-and-see” strategy of small firms may work towards cybersecurity underinvestment since they do not see themselves as being at high risk of cyberattacks.

4 The strengths of using the Tweedie model are threefold. The model efficiently examines the frequency and severity of losses by integrating the two into a single model (when p ∈ (1,2) as the compounding case) to address a highly (or extremely) right-skewed distribution. The Tweedie process as part of exponential dispersion models can be used with generalized linear models (GLM) (Jørgensen and de Souza, 1994). Finally, it can be used when data are scarce and there is a great number of no-claim (or loss) events (Peña-Sanchez 2019; Delong et al. 2021). The SAS database used in this study, as one of the largest operational risk databases, includes 37,652 observations, among which only 2852 (7.6% of the entire set) are cyber-related cases. This fact indicates that the scarcity of cyber risk-related data is a considerable challenge both in academia and in practice. Peña-Sanchez (2019) also points to the issue of data scarcity in the microinsurance context and suggests the Tweedie approach to overcome this challenge. Delong et al. (2021) employ an empirical insurance dataset in which claims are scarce to test the performance of the Tweedie model. We take advantage of these strengths by modelling cyber operational losses of the financial industry in this two-step approach.

5 For the estimation of the Tweedie model, we apply the canonical link function, which is defined as $$h\left(\mu \right)=\left\{\begin{array}{ll}\mathrm{log}\left(\mu \right), & \quad p=1 \\ {\mu }^{1-p}/(1-p), & \quad p>1\end{array}\right.$$, where $h\left(\mu \right)$ is the canonical link function and *p* is the power parameter of the Tweedie distribution (Ohlsson and Johansson, 2006). We implement the maximum likelihood estimation for canonical links to derive the GLM coefficients of the explanatory variables in the second step. For more detail on the model specification and statistical estimation, see Jørgensen (1987) and Ohlsson and Johansson (2006).

6 Wei et al. (2018) review 66 databases for operational risk and show that the SAS OpRisk database is the largest and most comprehensive database across all industries worldwide that is publicly available. Even larger, but not publicly available is the Operational Riskdata eXchange (ORX) database, which is a consortium-based database. Each of the operational risk databases have their pros and cons. On the one hand, in publicly reported data, firms may try to underreport the size of their losses or not report them at all. On the other hand, voluntary reporting between banks in consortium-based database is usually complete but foregoes some information on losses due to the anonymisation of the incidents (see Ganegoda and Evans 2013; Ames, Schuermann, Scott (2015)). The SAS database ensures strict data quality standards and periodic reviews; each loss event has been confirmed at least by one major media source and is thus traceable and peer-reviewable. This database has not only been applied in many academic papers (e.g., De Fontnouvelle et al. 2006; Hess 2011; Eling and Wirfs 2019) but is also widely used in practice (e.g., regulators allow banks and insurers to complement their own data with this dataset to calculate risk-based capital). Ames et al. (2015) argue that an external loss data such as that found in the SAS OpRisk database should be considered for operational loss modelling since it provides low-frequency-high-severity events that are otherwise rare to obtain, even for larger banks and insurers.

7 Large operational losses are of particular interest for financial firms because (1) such losses may prevent firms from meeting the regulatory requirement, and (2) those losses may place firms under pressure from stakeholders to strengthen their enterprise risk management schemes. This argument leads this study to focus on investigating the severity of large cyber losses. Ganegoda and Evans (2013) provide a systematic explanation to this approach for banks’ operational loss. The approach consists of (1) a base model selection for operational loss severities and (2) a regression analysis (generalized additive model in this paper) with the optimal base model for the target variable to examine its determinants.

8 For the search and identification strategy, we refer to Appendix B in Eling and Wirfs (2019), which states that three criteria (critical asset, actor and outcome) related to cyber risk should be met.

9 The focus of the SAS data collection is on the financial industry because these firms need such data for capital requirements. Additionally, the truncation at $100,000 does not pose a significant issue because we are primarily interested in loss events relevant for risk management and capital requirements.

10 In “Results” section, we also compare the estimates of risk measures for cyber operational losses and other operational losses. In Appendix 1, we show the summary statistics of variables associated with other operational loss events (Table 7) and the histograms of log-transformed operational losses in the financial and nonfinancial industries (Fig. 3). Comparing the cyber losses in Table 3 and Fig. 1, we observe that the losses of other operational risks are larger and more disperse than those of cyber risks; however, the distribution of log-transformed cyber losses tends to be more skewed. These differences are in line with Eling and Wirfs (2019), who document significant distance between the distributions of these two types of risks.

11 We take the key factors of the nested models to isolate the effects of such factors on the size of cyber costs.

12 The two GLM-based models are solely comparable cases to model a continuous response variable, while the three other univariate models are selected based on the evidence from the K-S tests shown in Table 4 or the empirical case.

13 We see this differentiation as critical to address the importance of considering heterogeneity in the level of interconnectivity and potential liability for the regulatory framework. Whether a loss amount was affected by the contagion risk and liability payment can be ex-post information related to a loss event; however, the finding that both factors consequentially have significant impacts on the loss size can imply that a heterogeneous level of interconnectivity and potential liability impact may need to be considered for the capital requirement.

14 We run the simulation with the R package tweedie, in which location, scale and power parameters that are calibrated with the fitted models are used as inputs.

15 This finding can be supported by Eisenbach et al. (2021), who analyse the spillover effects of cyber risk events in the U.S. financial industry. They find that the high level of interconnectivity found within the U.S. financial system can lead to high levels of vulnerability against a cyberattack.

16 Three key factors are considered when calculating the SMA capital needed, namely, buckets, the business indicator component (BIC) and the loss component (LC). Buckets consist of five categories and are determined by the size of the business indicator (BI) that is defined as the sum of the following three components: (1) the interest, lease and dividend component, (2) the services component and (3) the financial component (Peters et al. 2016). The BIC is a function of the BI and is differentiated by the above-mentioned buckets. The LC is calculated by a compound loss process, where weights are differentiated by the size of loss severity (i.e., between below and over € 100 million). Then, the SMA capital is determined in a simple manner; i.e., for firms that are categorized in Bucket 1, the capital is simply the BIC, while for others, the capital deviates from € 110 million depending on the BIC and the LC. We refer to Peters et al. (2016) and BIS (2016, Sect. 4) for the details of the SMA formulation.
